# The brown anole dewlap revisited: do predation pressure, sexual selection, and species recognition shape among-population signal diversity?

**DOI:** 10.7717/peerj.4722

**Published:** 2018-05-08

**Authors:** Simon Baeckens, Tess Driessens, Raoul Van Damme

**Affiliations:** 1 Laboratory of Functional Morphology, Department of Biology, University of Antwerp, Wilrijk, Belgium; 2 Museum of Comparative Zoology, Department of Organismic and Evolutionary Biology, Harvard University, Cambridge, MA, USA

**Keywords:** *Anolis sagrei*, Caribbean islands, Clay models, Dewlap diversity, Display behaviour, Lizards, Predation pressure, Visual signalling system

## Abstract

Animal signalling structures are amongst the most variable characteristics, as they are subjected to a diversity of selection pressures. A well-known example of a diverse signalling system in the animal kingdom is the dewlap of *Anolis* lizards. Dewlap characteristics can vary remarkably among and within species, and also between sexes. Although a considerable amount of studies have attempted to disentangle the functional significance of the staggering dewlap diversity in *Anolis*, the underlying evolutionary processes remain elusive. In this study, we focus on the contribution of biotic selective pressures in shaping geographic variation in dewlap design (size, colour, and pattern) and dewlap display behaviour at the intraspecific level. Notably, we have tried to replicate and extend previously reported results hereof in both sexes of the brown anole lizard (*Anolis sagrei*). To do this, we assembled a dataset consisting of 17 *A. sagrei* heterogeneous island populations from the Caribbean and specifically tested whether predation pressure, sexual selection, or species recognition could explain interpopulational variation in an array of dewlap characteristics. Our findings show that in neither males nor females estimates of predation pressure (island size, tail break frequency, model attack rate, presence of predatory *Leiocephalus* lizards) or sexual selection (sexual size dimorphism) could explain variation in dewlap design. We did find that *A. sagrei* males from larger islands showed higher dewlap display intensities than males from smaller islands, but the direct connection with predation pressure remains ambiguous and demands further investigation. Last, we could show indirect support for species recognition only in males, as they are more likely to have a ‘spotted’ dewlap pattern when co-occurring with a higher number of syntopic *Anolis* species. In conclusion, we found overall limited support for the idea that the extensive interpopulational variability in dewlap design and use in *A. sagrei* is mediated by variation in their biotic environment. We propose a variety of conceptual and methodological explanations for this unexpected finding.

## Introduction

Even more than most other animal traits, signalling structures are subjected to a diversity of selection pressures (Johnstone, 1997; Smith & Harper, 2003). To be effective, they have to be clear and conspicuous, often under a variety of environmental conditions (Endler, 1992). A single signalling structure is often used to convey different messages to multiple receivers, and therefore must be capable of reaching several sensing systems (Loyau, Jalme & Sorci, 2005; Finkbeiner, Briscoe & Reed, 2014). At the same time, any form of transmission is prone to eavesdropping by predators or parasites, and thus signalling structures should not be *too* prominent (Roberts et al., 2001; Clark, 2004; Bernal, Rand & Ryan, 2006). Adding to the complexity, as there are often multiple ways in which the same message (e.g. good genes) can be conveyed (e.g. by an acrobatic display, a bright-red crest, a particular odour), signalling structures also seem highly liable to the capriciousness of genetic drift (Richards-Zawacki, Yeager & Bart, 2013; Clark et al., 2015) and mate choice (Møller & Pomiankowski, 1993). It should not come as a surprise, then, that signalling structures are amongst the most variable animal characteristics (Zuk & Tinghitella, 2008), and that understanding their evolution has proved particularly challenging.

The dewlap, an extendible flap of skin attached to the throat, of *Anolis* lizards is no exception. Typically male, but also female, anoles display their often brightly coloured dewlap in a variety of contexts and the resultant signal is said to function in social and sexual communication (Greenberg & Noble, 1944; Jenssen, 1970; Crews, 1975; Carpenter, 1978), in species recognition (Rand & Williams, 1970; Losos, 1985), and in predator deterrence (Leal & Rodríguez-Robles, 1995, 1997a, 1997b). Dewlaps can differ greatly in size, shape, colour, and patterning, among species, among populations within species, and between sexes (Nicholson, Harmon & Losos, 2007). In 2009, Vanhooydonck and colleagues studied differences in dewlap characteristics among seven island populations of the brown anole (*Anolis sagrei*) from the Bahamas. They reported that dewlap pattern and size have evolved under different selection regimes. Notably, their data showed that diversity in dewlap pattern is best explained by the number of syntopic *Anolis* species (thus, *species recognition* as the hypothesised driving force), whereas variation in relative dewlap size is primarily explained by the presence or absence of predatory *Leiocephalus* lizards (*natural selection*) and to some extent by sexual size dimorphism (*sexual selection*; in males only). Relative dewlap size in males and females appeared to be larger on islands where *A. sagrei* occurred in sympatry with *Leiocephalus* lizards. Based on this finding, the authors suggested that the *A. sagrei* dewlap functions at least partly as a pursuit-deterrence signal.

In this study, we have tried to replicate Vanhooydonck’s results for both males and females using an extended dataset. To the data from the seven Bahamian islands (i.e. Acklins, Andros, Chub Cay, Crooked Island, Grand Bahama, Pidgeon Cay, Staniel Cay) reported in Vanhooydonck et al. (2009), we added *A. sagrei* populations from Cayman Brac, Cuba, Grand Cayman, Jamaica, Little Cayman, San Salvador, South Abaco, and South Bimini. Moreover, we measured two additional dewlap characteristics (dewlap colour and use) that have been suggested to play a critical role in anole diversification and speciation (Sigmund, 1983; Losos, 1985; Ord, Stamps & Losos, 2010; Macedonia et al., 2013; Ng et al., 2013, 2017). Using similar proxies for quantifying selective regimes as Vanhooydonck and co-workers (i.e. island size, tail break frequency (TBF), model attack rate, presence of predatory *Leiocephalus* lizards, number of syntopic *Anolis* species, sexual size dimorphism), we here test whether interpopulational variation in *A. sagrei* dewlap characteristics (design and display) can be explained by predation, species recognition, and/or sexual selection hypotheses.

## Materials and Methods

### Animals

**Figure 1:**
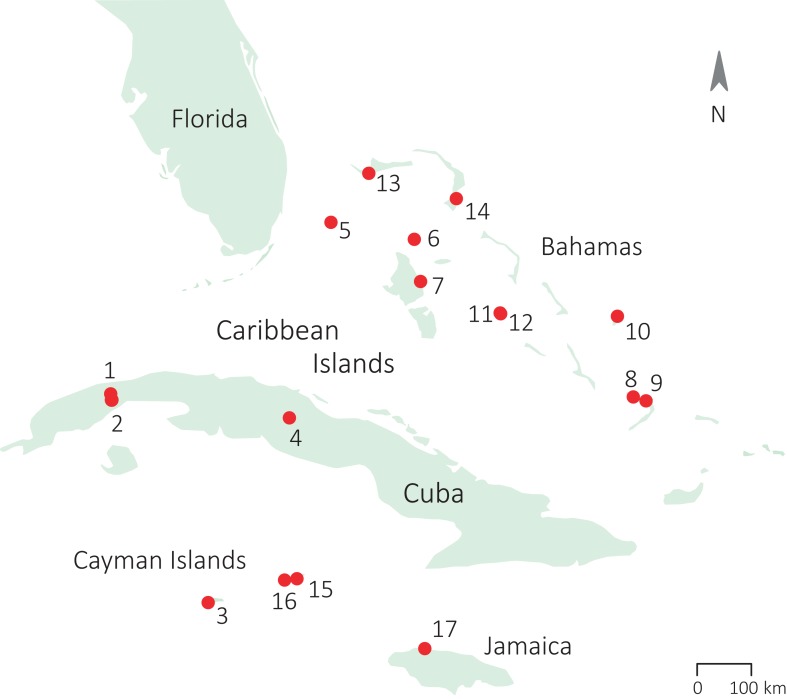
Sampling locations of the populations of study across the Caribbean. (1) Soroa (Cuba), population 1; (2) Soroa (Cuba) population 2; (3) Grand Cayman; (4) Santa Clara (Cuba); (5) South Bimini; (6) Chub Cay; (7) Andros; (8) Crooked Island; (9) Acklins; (10) San Salvador; (11) Staniel Cay; (12) Pidgeon Cay; (13) Grand Bahama; (14) South Abaco; (15) Cayman Brac; (16) Little Cayman; (17) Jamaica.

We collected data on adult *A. sagrei* lizards from nine populations in the Caribbean during the breeding seasons of 2012, 2013, and 2015 (March–September; Lee et al., 1989). Data on one additional population (San Salvador) was collected outside the breeding season (January 2013). Details on the sampling locations of our populations are provided in Fig. 1 and Table S1. Individuals were caught by noose and kept individually in plastic bags for maximum 48 h before being released back at the exact location of capture. We measured snout–vent length (SVL) with callipers (Mitutoyo CD-15DC, accuracy 0.01 mm) and quantified dewlap design for each captured lizard. We caught a total of 282 males and 245 females (raw data for 143 males and 117 females were kindly provided by Bieke Vanhooydonck from the study by Vanhooydonck et al., 2009). To quantify dewlap use, we observed the behaviour of another 235 males and 189 females. All work was carried out in accordance with the University of Antwerp animal welfare standards and protocol (ECD 2011-64) and the local environmental agencies (The Bahamas Environment, Science & Technology Commission, The Ministry of The Environment and Housing; Department of Environment Cayman Islands; Centre for Marine Science, University of the West Indies Jamaica).

### Dewlap measurements

#### Dewlap size

We used a technique outlined by Vanhooydonck et al. (2005) to estimate the surface area of the dewlap in every lizard caught. In short, we held the lizard on its left side against a 1 cm^2^ gridded paper and gently pulled the base of the ceratobranchial forward with a pair of forceps until the dewlap was fully extended parallel to the grid. We then photographed the dewlap, using a Nikon D70 camera mounted on a tripod. We used Adobe Photoshop CS3 extended software (AP CS3, version 10.0) to trace the outer edge of the dewlap on the digital images and to calculate absolute dewlap area for all lizards (17 populations). To remove effects of overall size, we regressed log_10_-transformed dewlap size against log_10_-transformed SVL (for males and females separately). The obtained unstandardized residuals of all individuals were then averaged per population and used as estimate of relative dewlap size.

#### Dewlap pattern

In most brown anoles, the alteration of red and yellow-coloured patches on the dewlap gives rise to a ‘pattern’ that can be categorised into three types (Nicholson, Harmon & Losos, 2007; Driessens et al., 2015). ‘Solid’ dewlaps are uniformly coloured; ‘marginal’ dewlaps have an evenly reddish coloured centre and a yellowish margin; and ‘spotted’ dewlaps have yellowish spots scattered across the reddish centre, regardless of the presence of a margin. One of us (T.D.) assigned each of the 425 male and 362 female dewlaps from the 17 study populations to one of the pattern categories on the basis of high-quality digital photos. We then determined the percentage of individuals attributed for the respective categories, per sex and per population.

#### Dewlap colour

We measured dewlap reflectance at the centre of the dewlap, using an Avantes spectrometer (AvaSpec-2048 USB2-UA-50; Avantes, Apeldoorn, the Netherlands, range 250–1000 nm) and deuterium-halogen light source (AvaLight-DHS; Avantes, Apeldoorn, the Netherlands) equipped with a fibre-optic probe. The probe was mounted within a metal holder to ensure readings at a fixed distance from the surface and was held perpendicular to the surface of the maximally extended dewlap. Reflectance data were collected for wavelengths from 300 to 700 nm, including the lower range of photon absorption by UV-sensitive photoreceptor cones published for anoles (Fleishman, Loew & Leal, 1993). To investigate dewlap colour variation, we interpolated each spectrum to 1 nm wavelength intervals and extracted four variables following Ng et al. (2013): brightness, hue, and relative reflectance in UV (RF 365 nm) and in red (RF 655 nm) (Montgomerie, 2006). We calculated brightness as the total area under the uncorrected spectral curve (300–700 nm) (Andersson, Örnborg & Andersson, 1998; Smiseth et al., 2001). For the remaining three colour variables, we corrected each spectrum for brightness by making the area under the curve equal to 1 (Endler, 1990). Hue was defined as the cut-on wavelength, i.e. the midpoint between baseline and maximum reflectance (Andersson, Örnborg & Andersson, 1998; Keyser & Hill, 2000; Saks, Mcgraw & Horak, 2003; Cummings, 2007). We decided to extract relative reflectance specifically in UV (365 nm) and red (655 nm), as the *A. sagrei* dewlap spectrum shows maxima and a high level of intraspecific variation at both wavelengths (Steffen & McGraw, 2007; T. Driessens, 2017, personal observation). Spectral measurements were carried out for 242 males and 217 females in total, distributed across nine populations. We do not have spectral data for the seven population sampled by Vanhooydonck et al. (2009), and for the population from Central Cuba (Santa Clara), due to technical problems with the spectrometer. All analyses of spectral data were run in R using the ‘pavo’ package (Maia et al., 2013).

#### Display behaviour

We observed each lizard (*N* = 20–30 males and *N* = 8–25 females per population; 10 populations in total) for 10 min in their natural environment, using a high definition camera (Sony, HDR-CX260VE). We first located lizards by walking silently through their natural habitat until an apparently undistributed individual was spotted. We then filmed the lizard from a distance using the camera zoom function in order to minimize any disturbance caused by our presence. Recordings were only made during sunny or partly clouded weather conditions and between 9 AM and 4:30 PM to avoid possible confounding effects of weather and time on the lizard’s activity level (Huey, 1982; Hertz, Huey & Stevenson, 1993). All behavioural recordings were scored offline, using the software JWatcher 1.0 (Blumstein, Evans & Daniel, 2000). We calculated the dewlap extension (DE) rate per individual (‘DE rate’); that is, the number of times the dewlap was extended per minute. We than calculated the average DE per population. Moreover, we calculated the proportion of individuals per population that were observed dewlapping at least once during the 10 min observation window (‘prop. DE’). Calculations were performed for each sex separately. We combined these two measures for displaying intensity into a single measure by feeding them into a principal component analyses (for each sex separately). The analysis produced a single component that explained >90% of the variation in males and females, and was highly positive correlated with DE rate and proportion DE. We used this combined measure (‘PC dewlap display’) to index the intensity of dewlap displaying behaviour in males and females of our study populations.

### Selection proxies

#### Predation pressure

**Figure 2:**
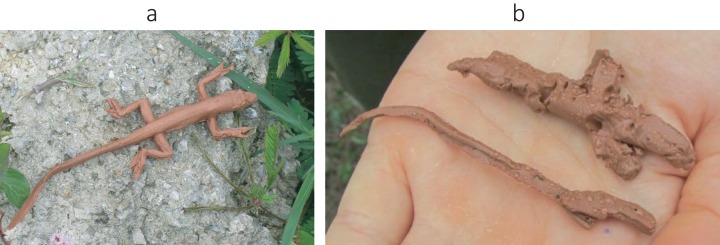
Clay model of a brown anole used for estimating predation pressure. Photograph of a clay *Anolis* model used for estimating predation pressure: (A) an intact model; (B) a recollected model with predation marks. Picture by Tess Driessens.

As in Vanhooydonck et al. (2009), we used island size, TBF, and the presence/absence of *Leiocephalus* lizards as proxies for predation pressure in the respective populations. Firstly, island size is a crude estimator of predation intensity, because larger islands tend to house larger numbers of predators, like raptors and snakes (Losos & Schluter, 2000; Ricklefs & Bermingham, 2004). Also, in the Bahamas, the survival rate of *A. sagrei* is substantially lower on larger islands with more bird species (Schoener & Schoener, 1982). Information on island area was obtained from the literature (Losos, Irschick & Schoener, 1994; Vanhooydonck et al., 2009; Bradley & Rey-Millet, 2013) or taken from websites (http://islands.unep.ch and http://www.geographia.com). Secondly, we concur with Vanhooydonck et al. (2009) and many other authors (Schoener & Schoener, 1980; Turner et al., 1982; Fox, Perea-Fox & Franco, 1994; Bateman & Fleming, 2009; Donihue et al., 2016; Itescu et al., 2017a, 2017b) that TBF can be a questionable measure of actual predation risk, and use it here for sake of conformity and in combination with other estimates of predation. This index was calculated as the ratio of the number of lizards with a regenerated tail to the total number of lizards captured for each population and separately per sex. Thirdly, the results of Vanhooydonck et al. (2009) suggest that the presence/absence of curly-tailed lizards (*Leiocephalus carinatus*) from their study islands in the Bahamas constitutes an important factor in the evolution of dewlap size. Curly-tailed lizards may exert their influence through competition for arthropod food or directly, by preying on anoles (Schoener, Slade & Stinson, 1982; Schoener, Spiller & Losos, 2002; Losos, Schoener & Spiller, 2004; Losos et al., 2006; López-Darias, Schoener & Spiller, 2012; Lapiedra, Chejanovski & Kolbe, 2017). For the smaller islands (<30 km^2^), we relied on data from the literature for deciding whether the *A. sagrei* populations were or were not syntopic with curly-tailed lizards. However, for the larger islands, we established a circle of 10 km radius (Dean, Smith & Engeman, 2004) around our study sites and considered them ‘under *Leiocephalus* pressure’ only when a curly-tailed lizard was seen within that area by us or by consulted local herpetologists. Finally, in addition to the three proxies of predation intensity used by Vanhooydonck et al. (2009), we tallied the number of clay models of *Anolis* lizards attacked by predators. This technique has been used successfully to estimate predation rate (especially by birds) in other anole studies (Brodie, 1993; Moore & Robinson, 2004; Husak et al., 2006; Steffen, 2009). We first constructed hundreds of models by pouring brown-coloured clay (Plastalina, Claytoon, Valencia, Spain) in a mould made from an *A. sagrei* specimen (Fig. 2A). On location, we placed approximately 120 clay models per sampling locality with a distance of 4–6 m in between. Models were randomly distributed on natural perches for *A. sagrei* lizards (trunk-ground ecomorph; Losos, 2009), including trunks, branches, stones, and litter on the ground. After leaving the sampling site undisturbed for 48 h, we recollected the clay models and scored for predator marks (i.e. clear attacks of birds, lizards and/or rodents, Fig. 2B). The proportion of attacked models was calculated as the number of recollected attacked models divided by the total number of recollected models, per population. Data on the proportion of attacked clay models could be collected for nine *A. sagrei* populations only.

#### Sexual selection

We used sexual size dimorphism (SSD) as a proxy for the intensity of sexual selection in each of our study populations. SSD has been widely used to gauge the strength of sexual selection in lizards, and anoles in particular, because there is strong evidence that a large body size increases a male’s competitiveness and thereby its access to females (Ord, Blumstein & Evans, 2001; Stuart-Fox & Ord, 2004; Ord & Martins, 2006; and references therein). Following Vanhooydonck et al. (2009), we here defined SSD as mean SVL in males divided by mean SVL in females, per population.

#### Species recognition

To test the ‘species recognition’ hypothesis, we noted the presence of any other *Anolis* species within our sampling areas, as in Vanhooydonck et al. (2009).

### Statistics

In this study, we considered interpopulational variation and therefore used population means and proportions of individuals per population as data points. We applied arcsine square root transformations to all proportion data (dewlap pattern, TBF, model attack rate) to meet normality assumptions (Sokal & Rohlf, 1995).

Our statistical analyses differed from the ones adopted by Vanhooydonck et al. (2009) in two important ways. Firstly, Vanhooydonck et al. (2009) adopted an information-theoretic approach to compare seven plausible models of selection for dewlap size and pattern. In doing so, they tested which combination of multiple predictor variables best describes the variation in dewlap characteristics. We are reluctant to take this approach because of the limited number of data points (seven populations in their case, nine to 17 in our extended dataset) relative to the number of predictors (see also Burnham & Anderson, 2002; Garamszegi, 2011). Rather, we opted for univariate regression analyses, linking individual predictor variables to individual dewlap characteristics. This allowed us to make full use of the information available for a particular pair of predictor and response variable (not all variables could be measured in all populations). Second, in analysing the data here, we took phylogenetic relationships among the study populations into account. The phylogeny used in the comparative analyses is the one proposed by Driessens et al. (2017), which is based on mtDNA haplotypes obtained by Kolbe et al. (2004). Phylogenetic regression analyses were conducted using the pgls() function in the ‘caper’ package in R (Revell, 2010; Orme et al., 2013). This method uses maximum likelihood to simultaneously estimate the regression model and the phylogenetic signal (Pagel’s λ) of the residual error (Garland & Ives, 2000; Revell, 2010). It has been shown to do better than a priori tests of phylogenetic signal to estimate the appropriateness of phylogenetically corrected tests, especially when sample sizes are smaller than 20 (Blomberg, Garland & Ives, 2003; Revell, 2010; Kamilar & Cooper, 2013). Comparisons of dewlap characteristics between islands with and without *Leiocephalus* lizards were conducted using the phylANOVA() function in the ‘phytools’ package in R (Revell, 2012). Because data from one population (San Salvador) could only be obtained outside the breeding season (see Materials and Methods, section ‘Animals’), we have run all analyses with and without inclusion of this population. Results were nearly identical and we will, therefore, report results for the complete dataset only. Raw *P*-values were corrected for multiple testing using the Benjamini–Hochberg (BH) procedure (Benjamini & Hochberg, 1995). All statistical analyses were conducted in R version 3.2.1 (R Development Core Team, 2016).

## Results

The raw data on dewlap characteristics can be found on Dryad (https://doi.org/10.5061/dryad.4572v), and the data on island size, TBF, model attack rate, presence/absence of curly-tailed lizards, SSD, and number of *Anolis* species, is presented in Table 1.

**Table 1:** Data on selection indices.

Population	Island size	TBF		Model attack rate	*L. carinatus*	SSD (*N*)	Number of *Anolis* species	*Anolis* species (excl. *A. sagrei*)
		Males (*N*)	Females (*N*)					
Acklins	310.8	0.55 (10)	0.09 (12)	–	1	1.43 (22)	2	*A. carolinensis*
Andros	5,957	0.50 (23)	0.45 (18)	–	0	1.23 (41)	4	*A. angusticeps*, *A. carolinensis*, *A. distichus*
Cayman Brac	38	0.29 (28)	0.10 (29)	0.008	1	1.33 (57)	2	*A. maynardi*
Chub Cay	15.76	0.35 (20)	0.35 (16)	–	1	1.32 (36)	4	*A. angusticeps*, *A. carolinensis*, *A. distichus*
Crooked Island	238.28	0.46 (23)	0.48 (20)	–	1	1.25 (43)	2	*A. carolinensis*
Grand Bahama	1,373	0.56 (24)	0.38 (11)	–	1	1.33 (35)	3	*A. carolinensis*, *A. distichus*
Grand Cayman	197	0.26 (27)	0.10 (29)	0.070	1	1.28 (56)	2	*A. conspersus*
Jamaica	10,911	0.29 (32)	0.29 (23)	0.073	0	1.24 (55)	3	*A. lineatopus*, *A. grahami*
Little Cayman	28	0.59 (28)	0.43 (27)	0.034	1	1.29 (55)	2	*A. maynardi*
Pidgeon Cay	0.019	0.47 (16)	0.25 (8)	–	0	1.21 (24)	2	*A. carolinensis*
San Salvador	163	0.41 (27)	0.48 (14)	0.067	1	1.35 (41)	2	*A. distichus*
Santa Clara	105,006	0.67 (27)	0.58 (24)	0.020	0	1.33 (51)	2	*A. allisoni*
Soroa 1	105,006	0.42 (23)	0.38 (21)	–	0	1.24 (44)	3	*A. homolechis*, *A. porcatus*
Soroa 2	105,006	0.50 (22)	0.46 (24)	0.019	0	1.32 (46)	3	*A. homolechis*, *A. porcatus*
South Abaco	1,145.9	0.30 (26)	0.29 (21)	0.008	1	1.28 (47)	2	*A. smaragdinus*
South Bimini	10.36	0.44 (27)	0.36 (23)	0.000	1	1.30 (50)	4	*A. angusticeps*, *A. carolinensis*, *A. distichus*
Staniel Cay	5.18	0.37 (26)	0.33 (20)	–	0	1.32 (46)	3	*A. carolinensis*, *A. distichus*

**Notes:**

Island size, tail break frequency (TBF) for males and females, proportion of attacked clay models, presence/absence of *Leiocephalus carinatus* lizards (0 = absent, 1 = present), sexual size dimorphism (SSD), and total number of co-occurring *Anolis* species; ‘–’ represents missing data. Sample sizes (*N*) used to calculate TBF and SSD are also provided.

None of the four measures assumed to index the intensity of predation in our study populations correlated significantly with any of the dewlap design characteristics (Table 2, all *P* > 0.06). Dewlap size, colour, and pattern also did not differ consistently between populations syntopic or not with the predatory curly-tailed lizard (Table 2, all *P* > 0.13). These results suggest that interpopulational differences in dewlap design may not be driven by differences in predation intensity. We did find evidence for an effect of island size on dewlap display behaviour in males. Male anoles on larger islands scored higher on PC dewlap display, indicating that they used their dewlap more often than conspecifics on smaller islands (*P* = 0.002). All other proxies for predation pressure (TBF, model attack rate, presence/absence of curly-tailed lizards) did not have a comparable effect on male display intensity (all *P* > 0.15). Neither did we find any relationship between predation intensity and female display rate (all *P* > 0.20).

**Table 2:** Data on predation pressure.

Predation pressure															
Dewlap variables	Island size				Tail break frequency (TBF)				Model attack rate				*L. carinatus*		
	*N*	*b*	*SE*	*P*	*N*	*b*	*SE*	*P*	*N*	*b*	*SE*	*P*	*N*	*F*	*P*
**Males**															
Relative dewlap size	17	3.7 × 10^−7^	4.2 × 10^−7^	0.392	17	0.241	0.131	0.086	9	−0.038	0.237	0.877	17	0.678	0.461
Pattern ‘solid’	17	−1.8 × 10^−6^	2.6 × 10^−6^	0.498	17	0.757	0.569	0.204	9	1.684	1.009	0.139	17	0.686	0.457
Pattern ‘marginal’	17	5.5 × 10^−8^	2.5 × 10^−6^	0.982	17	−0.587	0.525	0.281	9	1.475	1.411	0.331	17	0.004	0.961
Pattern ‘spotted’	17	1.5 × 10^−6^	2.4 × 10^−6^	0.558	17	−0.104	0.539	0.850	9	−3.006	1.183	0.351	17	0.779	0.458
Colour brightness	9	1.4 × 10^−2^	9.9 × 10^−3^	0.197	9	−2405	4044	0.571	8	−3709	4880	0.476	9	0.422	0.575
Colour hue	9	−5.0 × 10^−5^	4.2 × 10^−5^	0.275	9	−14.88	12.08	0.257	8	−2.11	22.03	0.927	9	3.545	0.135
Colour RF365 nm	9	−3.6 × 10^−9^	2.1 × 10^−9^	0.135	9	−7.3 × 10^−4^	6.0 × 10^−4^	0.261	8	−1.3 × 10^−4^	1.2 × 10^−3^	0.917	9	2.979	0.137
Colour RF655 nm	9	2.2 × 10^−9^	3.9 × 10^−9^	0.592	9	9.6 × 10^−4^	1.1 × 10^−3^	0.409	8	1.1 × 10^−3^	1.8 × 10^−3^	0.571	9	3.088	0.147
PC dewlap display	**10**	**1.8** × **10**^−**5**^	**2.9** × **10**^−**6**^	**0.002**	10	2.587	1.656	0.157	9	−2.203	3.360	0.533	10	2.277	0.210
**Females**															
Relative dewlap size	17	4.7 × 10^−7^	2.8 × 10^−7^	0.114	17	−0.002	0.073	0.980	9	−0.254	0.153	0.140	17	0.018	0.920
Pattern ‘solid’	17	−4.8 × 10^−6^	3.0 × 10^−6^	0.131	17	−0.946	0.548	0.105	9	−0.506	1.381	0.725	17	0.004	0.950
Pattern ‘marginal’	17	5.4 × 10^−6^	2.7 × 10^−6^	0.062	17	1.135	0.461	0.176	9	1.693	1.251	0.218	17	0.106	0.746
Pattern ‘spotted’	17	−1.9 × 10^−7^	1.8 × 10^−6^	0.916	17	0.253	0.437	0.571	9	−1.689	0.967	0.124	17	0.636	0.487
Colour brightness	9	6.1 × 10^−3^	1.0 × 10^−2^	0.577	9	−5349	2112	0.176	8	−4188	5199	0.451	9	0.322	0.633
Colour hue	9	−3.9 × 10^−5^	4.9 × 10^−5^	0.455	9	−3.61	13.18	0.792	8	18.18	25.05	0.495	9	3.421	0.113
Colour RF365 nm	9	−3.6 × 10^−9^	2.0 × 10^−9^	0.115	9	−6.7 × 10^−4^	4.1 × 10^−4^	0.146	8	−5.1 × 10^−5^	1.1 × 10^−3^	0.965	9	0.924	0.399
Colour RF655 nm	9	7.1 × 10^−9^	2.8 × 10^−9^	0.236	9	1.2 × 10^−3^	6.6 × 10^−4^	0.120	8	2.2 × 10^−4^	1.8 × 10^−3^	0.907	9	0.459	0.555
PC dewlap display	10	8.8 × 10^−6^	6.4 × 10^−6^	0.204	10	0.846	1.955	0.676	9	−2.285	4.065	0.591	10	0.040	0.877

**Notes:**

Univariate pgls regression analyses of dewlap design and display versus estimates of predation intensity (island size, tail break frequency, and model attack rate). Phylogenetic analyses of variance incorporation dewlap design and use versus presence or absence of predatory curly-tailed lizards. Results are shown separately per sex; *b* indicates the regression coefficient and SE, its standard error. Significant results (BH-corrected *P-*value) are in bold. See ‘Statistics’ section for more details.

In neither males nor females, differences in SSD significantly contributed to interpopulational variation in dewlap characteristics (Table 3, all *P* > 0.11). In populations exhibiting stronger size dimorphism, males nor females had dewlaps that were consistently larger, brighter, or of a different hue than in populations with limited SSD. Nor did they have dewlaps that reflected more in the UV or red region. We found also no evidence for a relationship between SSD and the proportion of different types of dewlap patterns (i.e. solid, marginal, or spotted). Together, these findings do not suggest that differences in the intensity of sexual selection among the populations contribute to among-island variation in dewlap design.

**Table 3:** Sexual selection and species recognition.

	Sexual selection				Species recognition			
Dewlap variables	Sexual size dimorphism (SSD)				Number of *Anolis* species			
	*N*	*b*	*SE*	*P*	*N*	*b*	*SE*	*P*
**Males**								
Relative dewlap size	17	−0.014	0.323	0.966	17	0.011	0.022	0.632
Pattern ‘solid’	17	−0.937	1.178	0.439	17	−0.081	0.134	0.554
Pattern ‘marginal’	17	−0.250	1.094	0.822	17	−0.168	0.115	0.168
Pattern ‘spotted’	17	−0.984	1.035	0.357	**17**	**0.471**	**0.094**	**0.001**
Colour brightness	9	−1,0549	12,179	0.415	9	564.9	652.9	0.416
Colour hue	9	67.45	39.45	0.131	9	−2.552	2.617	0.362
Colour RF365 nm	9	0.001	0.002	0.701	9	1.1 × 10−^4^	1.4 × 10^−4^	0.473
Colour RF655 nm	9	−0.001	0.003	0.741	9	−2.2 × 10^−4^	2.4 × 10^−3^	0.388
PC dewlap display	10	8.327	5.290	0.154	10	0.243	0.421	0.580
**Females**								
Relative dewlap size	17	−0.026	0.211	0.907	17	0.015	0.015	0.349
Pattern ‘solid’	17	1.354	1.641	0.422	17	0.076	0.185	0.687
Pattern ‘marginal’	17	−0.677	1.696	0.696	17	0.089	0.171	0.609
Pattern ‘spotted’	17	−0.447	1.324	0.740	17	0.084	0.089	0.362
Colour brightness	9	−6,202	12,159	0.626	9	63.93	664.9	0.926
Colour hue	9	65.08	49.10	0.227	9	−3.250	3.000	0.314
Colour RF365 nm	9	−0.001	0.002	0.751	9	7.9 × 10^−5^	1.4 × 10^−4^	0.595
Colour RF655 nm	9	0.003	0.003	0.401	9	−6.0 × 10^−5^	2.4 × 10^−4^	0.811
PC dewlap display	10	3.462	8.755	0.703	10	−0.257	0.486	0.612

**Notes:**

Univariate pgls regression analyses of dewlap design and display versus sexual size dimorphism (SSD) and total number of co-occurring *Anolis* species. Results are shown separately per sex; *b* indicates the regression coefficient and SE, its standard error. Significant results (BH-corrected *P-*value) are in bold. See ‘Statistics’ section for more details.

Finally, we found no significant relationship between the number of co-occurring *Anolis* species and relative dewlap size, brightness, hue, or reflectance in UV or red regions (Table 3, all *P* > 0.31). We neither found an effect on dewlap display behaviour (*P* > 0.58). Interestingly, males—but not females—of populations with higher numbers of syntopic congeneric species were more likely to have a ‘spotted’ dewlap pattern (*P* = 0.001 in males).

Running standard (traditional) regression analyses without the incorporation of phylogenic relationships revealed similar results.

## Discussion

Overall, we found limited support for the idea that the extensive among-population variability in dewlap characteristics in *A. sagrei* is mediated by variation in their biotic environment. SSD, an index of sexual selection, varied considerably among our study populations, but did not correlate with any of the structural aspects of the dewlap considered, or with the intensity of displaying behaviour. Of the four proxies we used to assess relative predation intensity, none explained differences in dewlap design, and only one (i.e. island size) was associated with increased dewlap use. Our results do corroborate the hypothesis that the complexity of dewlap patterning functions in species recognition—at least in males. The relative size or colour characteristics of the dewlap, however, did not change consistently with the number of co-occurring congeneric species.

Vanhooydonck et al. (2009), using information on a subset of the populations studied here, concluded that predation pressure, especially the presence of predatory *L. carinatus* lizards, plays a significant role in the evolution of relative dewlap size. They argued that anoles may evolve larger dewlaps when in syntopy with these saurophagous lizards, because a large size would benefit the pursuit deterrence function of the dewlap. The predator deterrence hypothesis holds that prey performs eye-catching displays to warn the predator that its presence has been detected and that pursuits are likely to be futile or even dangerous (Hasson, 1991). It is a well-established fact that many lizard species will indeed engage in conspicuous displaying behaviour when confronted with a predator (reviewed in Greene, 1988). However, in most cases, these displays involve tail vibrations, curling or waving (although not that common in anoles as in other lizard groups; Dial, 1986; Hasson, Hibbard & Ceballos, 1989; Cooper, 2001, 2007, 2010, 2011; Telemeco, Baird & Shine, 2011; York & Bairds, 2016), and they are more likely intended to deflect the predator’s attack towards less vulnerable, expendable body parts, rather than to discourage pursuit. Leal & Rodríguez-Robles (1995, 1997a, 1997b) have argued that dewlapping in *A. cristatellus* and *A. cuvieri* may act as a pursuit deterrence signal, but the evidence is weak. In laboratory conditions, specimens of *A. cristatellus* were reported to dewlap ‘only rarely’ when a live native snake predator was introduced into their cage (Leal & Rodríguez-Robles, 1995); in the field, specimens of the same species did not extend their dewlaps more often when a snake model was moved into their territory (Leal & Rodríguez-Robles, 1997a). In their paper on *A. cuvieri*, Leal & Rodriguez-Robles (1997b) reported DEs in response to a snake model in only one individual out of a total of five. Moreover, several recent studies on *A. sagrei* found no evidence for increased DE rates in response to predatory birds (Simon, 2007; Elmasri et al., 2012), snakes (Yee et al., 2013), or curly-tailed lizards (Driessens, Vanhooydonck & Damme, 2014; Steinberg et al., 2014). For these reasons, we are sceptical about the role of the dewlap as a pursuit-deterrent and, hence, about predation pressure as a driver for dewlap size evolution. Accordingly, our analyses show very little evidence for a link between the used proxies of predation pressure and dewlap size—or any other structural aspect of the dewlap.

We are fully aware of the difficulty of measuring predation pressure. Each of the methods we employed has been criticized. Firstly, tail break frequencies may reflect predator inefficiency, rather than intensity (Schoener & Schoener, 1980; Turner et al., 1982; Jaksic & Greene, 1984; Fox, Perea-Fox & Franco, 1994). Secondly, stationary clay models do not adequately mimic natural organisms with respect to traits such as odour, anti-predator postures, or movement (Rangen, Clark & Hobson, 2000; Thompson & Burhans, 2004; Cooper, Caldwell & Vitt, 2008; Santos & Cannatella, 2011; Paluh, Hantak & Saporito, 2014). Moreover, our model attack rates estimated especially predation by birds, but other predators like snakes and lizards can impose high predation threats as well (Schoener, Slade & Stinson, 1982; Henderson & Crother, 1989; Rodríguez-Robles, 1992; Rodríguez-Cabrera et al., 2016). Thirdly, island size and the presence/absence of *L. carinatus* can be considered as very crude estimates of predator pressure—at best. On the other hand, some esteemed studies have established curly-tailed lizards as important drivers of morphological and behavioural diversification in *A. sagrei* (Losos, Schoener & Spiller, 2004; Losos et al., 2006; Lapiedra, Chejanovski & Kolbe, 2017). Lastly, prompting even more caution, none of the four respective proxies of predation intensity used in this study varied in concert (correlation analyses, all *R* < 0.22 and all *P* > 0.24). Perhaps the number of predatory species present on each island may provide more accurate information on the role of predation pressure in shaping dewlap design. Yet, greater species richness does not necessarily translate into higher predation rates as each predatory species may be less abundant or may include anoles as a smaller part of the diet (Losos, 2009). Combining the total number of predatory species with measures of their abundance and diet composition might be most appropriate, but is hardly feasible in the field when surveying multiple sites in a short period of time. Admittedly, an accurate quantification of predation pressure in the field is very challenging. Our findings that predation pressure does not contribute to the evolution of dewlap design in the brown anole lizard across the islands included in our study remains therefore highly tentative and demands further research.

For dewlap displays, we did find that *A. sagrei* males from larger islands showed higher display intensities than males from smaller islands. Yet, part of this relationship might be driven by the artefact that the three populations sampled on Cuba (three times the same ‘large’ value for island size) exhibited high display rates. Besides, it is highly questionable whether the positive correlation between island size and male display rate is truly because larger islands harbour more predators. Island size is known to influence many ecologically relevant variables (e.g. habitat complexity, community richness; Ricklefs & Lovette, 1999; Losos & Schluter, 2000) that were not considered here. Future studies are required to clarify and interpret our result showing that males use dewlaps more on large islands (i.e. non-independent island size data points from Cuba, islands size as accurate index of predation pressure).

The model that best explained the variation in male relative dewlap size in Vanhooydonck et al.’s dataset also contained sexual selection (SSD) as a predictor variable: on islands with high SSD (assumed to reflect high intensity of sexual selection), males tended to have larger dewlaps (Vanhooydonck et al., 2009). However, in our extended dataset, we found no indication that differences in SSD among islands are reflected in relative dewlap size, or any other dewlap characteristic. We can think of three ways to explain this result. First, sexual selection is simply not acting on dewlap traits. This sounds highly improbable, because the dewlap has all the characteristics of a sexually secondary trait: it is highly dimorphic in adult individuals, and exhibits the typical developmental pattern with sex-specific growth trajectories once the age of maturity is reached (Vanhooydonck et al., 2015). Several studies have shown that male brown anoles use their dewlap during territorial disputes (Scott, 1984) and/or during courtship (Simon, 2011; Driessens, Vanhooydonck & Damme, 2014). At least in males, aspects of the dewlap reveal information on the individual signalling that is highly relevant in a sexual selection context (Vanhooydonck et al., 2005; Driessens et al., 2015). Second, the effect of differences in the intensity of sexual selection among populations may be offset or overruled by some other factor. Natural selection may be counterbalancing or constraining any effects that divergent sexual selection is having on the among-island variation in dewlap characteristics (but see above). Dewlap traits may also be under differential selection for reasons not considered here. For instance, the climatic conditions and structural habitats in which our study populations live vary considerably. Physical aspects of the environment have been shown to influence the effectiveness of signals to a great extent in several animal species (Hebets & Papaj, 2005; Stuart-Fox, Moussalli & Whiting, 2007; Baeckens et al., 2018), including anoles (Leal & Fleishman, 2004; Ord et al., 2007; Ng et al., 2013). Moreover, Driessens et al. (2017) established that in *A. sagrei*, the dewlap of brown anoles occurring in ‘xeric’ environments differ from those inhabiting ‘mesic’ environment in dewlap colour, pattern, and use. They argue that the strong relationship between signal design and prevailing environmental conditions might result from differential selection on signal efficacy. A third explanation for the apparent lack of a relationship between sexual selection and dewlap design is methodological: SSD may simply not be a good proxy for the intensity of sexual selection. Although male-biased SSD in lizards is traditionally linked to intrasexual selection (Trivers, 1976; Stamps, 1983, 1999; Stamps, Losos & Andrews, 1997; Ord, Blumstein & Evans, 2001), evolutionary shifts in male aggression, territoriality and (relative) home range size explain but a small proportion of evolutionary changes in SSD (Cox, Skelly & John-Alder, 2003), suggesting that other factors may be at play. Recently, Bonneaud et al. (2016) presented evidence that sex-specific developmental plasticity may contribute to adult SSD in brown anoles: on six Bahamian islands, the amount of food biomass explained variation in male, but not female body size, giving rise to significant differences in SSD. Clearly, future studies should invest in collecting information on more reliable estimates of the intensity of sexual selection.

Our analyses lend partial support to the finding that dewlap ‘patterning’ may play a role in species recognition. Male brown anoles are more likely to have a ‘spotted’ dewlap when co-occurring with several other *Anolis* species. Whereas Vanhooydonck et al. (2009) found this to be true in both sexes, the effect was only significant in males in our extended dataset. Perhaps males that can broadcast their species identity properly are less likely to be attacked by non-conspecific males. Indeed, male anoles can behave very aggressively towards conspecific males, while at the same time ignoring males of other *Anolis* species (Losos, 1985). Females, on the other hand, may communicate their species identity in some other way (perhaps via head bobbing patterns).

Rand & Williams (1970) coined the species recognition hypothesis for explaining dewlap diversity in *Anolis* five decades ago. Subsequent behavioural studies on pairs of anole species have offered further support to the hypothesis (Losos, 1985; Macedonia & Stamps, 1994; Macedonia et al., 2013), but a broad-scale, phylogenetically informed analysis could not provide statistical corroboration (Nicholson, Harmon & Losos, 2007). In this study, we only hold qualitative information based on personal observations and data from the literature to speculate on how the species recognition hypothesis might explain the success of a ‘spotted’ dewlap pattern in *A. sagrei* males when co-occurring with a high number of other *Anolis* species. As the species recognition hypothesis predicts that sympatric species will have different dewlap configurations (Losos & Chu, 1998), we expect the spotted dewlap pattern of *A. sagrei* to be unique in places where many *Anolis* species occur. Indeed, the dewlap patterning of the majority of syntopic species we observed is described as solid (*A. allisoni, A. angusticeps, A. carolinensis, A. conspersus, A. homolechis, A. maynardi, A. porcatus*) or marginal (*A. grahami*) (Nicholson, Harmon & Losos, 2007; Vanhooydonck et al., 2009), which strengthens the idea that a spotted dewlap might function as a species recognition signal in *A. sagrei*. Two other syntopic species (*A. distichus* and *A. lineatopus*), however, are known to vary in their dewlap patterning among populations (Nicholson, Harmon & Losos, 2007; Ng et al., 2013), and we do not have the data to confirm whether they have a non-spotted dewlap when co-occuring with *A. sagrei*. The brown anole seems clearly an interesting study animal to deeper explore the possibility of sexual character displacement, but this will require detailed data on the signals of the co-occurring species and behavioural experiments.

## Conclusion

Using a comparative approach, and based on estimates of predation pressure, sexual selection, and species recognition, we find limited evidence for the hypothesis that the vast among-population variability in dewlap characteristics in *A. sagrei* is driven by variation in their biotic environment. In this work, we offer a number of explanations for this unexpected finding and stress that this outcome might also be (partially) attributed to our study design, in specific, the choice and number of study islands. The initial study by Vanhooydonck et al. (2009) focused on a (although small) set of relatively homogeneous Bahamian islands (i.e. small variation in biotic features, such as, island size, number of conspecifics, predation pressure); in contrast, our extended dataset includes some ‘mega-islands’ (such as Cuba and Jamaica) and non-native populations (Grand Cayman, Jamaica; Kolbe et al., 2004), which might obscure clear patterns of dewlap variation. Moreover, although our comprehensive dataset encompasses over 600 lizards from 17 different populations, an increase in sample size would have strengthened our findings. Although sample size affects science in general, if correlations are weak or non-significant (as many in this study), regression analyses typically demand high sample sizes to reach high statistical power. Therefore, we encourage further research on the role of the biotic environment in shaping the intraspecific diversity in dewlap design. Ideally, comparative studies should include more similar-sized island populations from the brown anole’s native range (e.g. only focussing on islets in the Bahamas), or scholars should opt for an experimental approach with a controlled and replicated study design in natural populations (as in, e.g. Losos, Schoener & Spiller, 2004; Calsbeek & Cox, 2010).

## Supplemental Information

10.7717/peerj.4722/supp-1 Supplemental Information 1 Islands of study. Sampling locations of the populations of study across the Caribbean (figure and exact geographic coordinates).

## Additional Information and Declarations

### Field Study Permissions

The following information was supplied relating to field study approvals (i.e. approving body and any reference numbers): Field experiments were approved by the local environmental agencies (The Bahamas Environment, Science & Technology Commission; The Ministry of The Environment and Housing; Department of Environment Cayman Islands; Centre for Marine Science, University of the West Indies Jamaica).

### Data Availability

The following information was supplied regarding data availability: The raw data are included in Tables 1–3.
