# Supplementary material for: The brown anole dewlap revisited: do predation pressure, sexual selection, and species recognition shape among-population signal diversity?
Source: PeerJ. 2018 May 8;6:e4722. doi: 10.7717/peerj.4722 (PMC5947042; doi:10.7717/peerj.4722)
Supplement: Supplemental Information 1 — Sampling locations of the populations of study across the Caribbean (figure and exact geographic coordinates). [file peerj-06-4722-s001.docx]

Supplemental Information

**Table S1** — Geographical coordinates of sampling locality are provided for each study population.

| Population | Coordinates |
| --- | --- |
|  |  |
| Acklins | 22°41'22.61" N — 73°58'55.49" W |
| Andros | 24°42'16.71" N — 77°47'14.51" W |
| Cayman Brac | 19°41'11.37" N ­— 79°52'27.18" W |
| Chub Cay | 25°24'59.56" N — 77°53'35.84" W |
| Crooked Island | 22°45'18.88" N — 74°11'48.19" W |
| Grand Bahama | 26°31'50.77" N — 78°39'41.62" W |
| Grand Cayman | 19°17'02.03" N — 81°22'24.33" W |
| Jamaica | 18°30'24.81" N — 77°42'58.25" W |
| Little Cayman | 19°39'50.58" N — 80° 05'0.39" W |
| Pidgeon Cay | 24°09'35.17" N — 76°26'06.71" W |
| San Salvador | 24°07'02.60" N — 74°27'52.23" W |
| Santa Clara | 22°24'08.81" N — 80° 00'06.95" W |
| Soroa 1 | 22°48'26.60" N — 83°01'06.60" W |
| Soroa 2 | 22°46'40.00" N — 83°00'07.70" W |
| South Abaco | 26°06'12.80" N — 77°11'02.00" W |
| South Bimini | 25°42'11.80" N — 79°18'00.50" W |
| Staniel Cay | 24°10'22.40" N — 76°26'40.98" W |
